# The sensor kinase BfmS controls production of outer membrane vesicles in *Acinetobacter baumannii*

**DOI:** 10.1186/s12866-019-1679-0

**Published:** 2019-12-21

**Authors:** Se Yeon Kim, Mi Hyun Kim, Seung Il Kim, Joo Hee Son, Shukho Kim, Yoo Chul Lee, Minsang Shin, Man Hwan Oh, Je Chul Lee

**Affiliations:** 1 Department of Microbiology, School of Medicine, Kyungpook National University, 680 Gukchaebosang-ro, Jung-gu, Daegu, 41944 Republic of Korea; 2 Drug & Disease Target Team, Korea Basic Science Institute, Ochang, Republic of Korea; 3 Department of Bio-Analytical Science, University of Science and Technology (UST), Daejeon, Republic of Korea; 4 Department of Nanobiomedical Science, Dankook University, 119 Dandae-ro, Dongnam-gu, Cheonan, Chungnam 31116 Republic of Korea

**Keywords:** *Acinetobacter baumannii*, BfmS, Cytotoxicity, OmpA, Outer membrane vesicle

## Abstract

**Background:**

*Acinetobacter baumannii* is an important opportunistic pathogen responsible for various nosocomial infections. The BfmRS two-component system plays a role in pathogenesis and antimicrobial resistance of *A. baumannii* via regulation of bacterial envelope structures. This study investigated the role of the sensor kinase, BfmS, in localization of outer membrane protein A (OmpA) in the outer membrane and production of outer membrane vesicles (OMVs) using wild-type *A. baumannii* ATCC 17978, Δ*bfmS* mutant, and *bfmS*-complemented strains.

**Results:**

The Δ*bfmS* mutant showed hypermucoid phenotype in the culture plates, growth retardation under static culture conditions, and reduced susceptibility to aztreonam and colistin compared to the wild-type strain. The Δ*bfmS* mutant produced less OmpA in the outer membrane but released more OmpA via OMVs than the wild-type strain, even though expression of *ompA* and its protein production were not different between the two strains. The Δ*bfmS* mutant produced 2.35 times more OMV particles and 4.46 times more OMV proteins than the wild-type stain. The Δ*bfmS* mutant OMVs were more cytotoxic towards A549 cells than wild-type strain OMVs.

**Conclusions:**

The present study demonstrates that BfmS controls production of OMVs in *A. baumannii*. Moreover, BfmS negatively regulates antimicrobial resistance of *A. baumannii* and OMV-mediated host cell cytotoxicity. Our results indicate that BfmS negatively controls the pathogenic traits of *A. baumannii* via cell envelope structures and OMV production.

## Background

*Acinetobacter baumannii* is a clinically important opportunistic pathogen responsible for various nosocomial infections, including ventilator-associated pneumonia, bacteremia, skin and soft tissue infections, urinary tract infections, and meningitis, especially in critically ill patients [1–3]. Treatment of this microorganism is challenging due to antimicrobial resistance, particularly to carbapenems and colistin [4, 5]. *A. baumannii* is one of the ‘ESKAPE’ pathogens, which are potentially antimicrobial resistant bacteria [6]. Despite their growing clinical importance, the pathogenic mechanisms of *A. baumannii* remain to be characterized. Of the identified virulence factors, outer membrane protein A (OmpA) is the most abundant outer membrane protein and plays a role in the pathogenesis of *A. baumannii* infections through biofilm formation, outer membrane vesicle (OMV) production, adherence and invasion in host cells, inactivation of the complement cascade, and host cell death [7–14]. In addition, OmpA is a major protein component in *A. baumannii* OMVs, in which OmpA contributes to host cell cytotoxicity and innate immune responses [13, 15]. OmpA production is tightly regulated by posttranscriptional ribo-regulation in *Escherichia coli* [16]. The production of OmpA is dependent on bacterial growth rate and is controlled by many environmental stresses [16–18]. However, little is known about the mechanisms that control localization of OmpA in either the outer membrane or OMVs.

Bacterial two-component systems (TCSs) are key factors that regulate virulence and antimicrobial resistance, and bacterial adaptation and survival in response to environmental stimuli [19, 20]. TCSs consist of a sensor kinase that senses extracellular or intracellular stimuli embedded in the cytoplasmic membrane, and a response regulator that relays signals in the cytoplasm [21]. The response regulator is a transcription factor that undergoes a conformational change upon phosphorylation and facilitates DNA binding. In *A. baumannii*, BfmRS regulates cell envelope structures important for virulence and antimicrobial resistance [22, 23]. The response regulator BfmR controls expression of the K locus that harbors genes for exopolysaccharide production and expression of the *csuA/BABCDE* operon for pili production [22, 24]. The Δ*bfmR* mutant showed complete loss of biofilm formation, reduced survival in human ascitic fluid and serum, and increased susceptibility to certain antimicrobial agents [24–27], whereas the Δ*bfmS* mutant exhibited enhanced virulence via hyperproduction of exopolysaccharides [22, 23], suggesting that BfmS negatively regulates its cognate response regulator BfmR. However, other studies demonstrated that Tn-inserted *bfmS* mutants showed a reduction in surface motility and bacterial growth in *Galleria mellonella* larvae [28, 29]. Interestingly, one previous study demonstrated that the BfmS-deficient mutant increasingly released OmpA, TEM-1 β-lactamase, and CarO into the supernatant compared to the wild-type *A. baumannii* strain [30]. This observation suggests that BfmS possibly controls production of OMVs, because a large amount of OmpA in culture supernatant is found in OMVs [13]. The present study was conducted to investigate whether sensor kinase BfmS controls localization of OmpA in either the outer membrane or OMVs, which subsequently affects OMV production, using wild-type *A. baumannii* ATCC 17978, Δ*bfmS* mutant, and *bfmS*-complemented strains.

## Results

### Low production of OmpA in the outer membrane of *A. baumannii* mutant with Tn-inserted *bfmS* gene

**Fig. 1:**
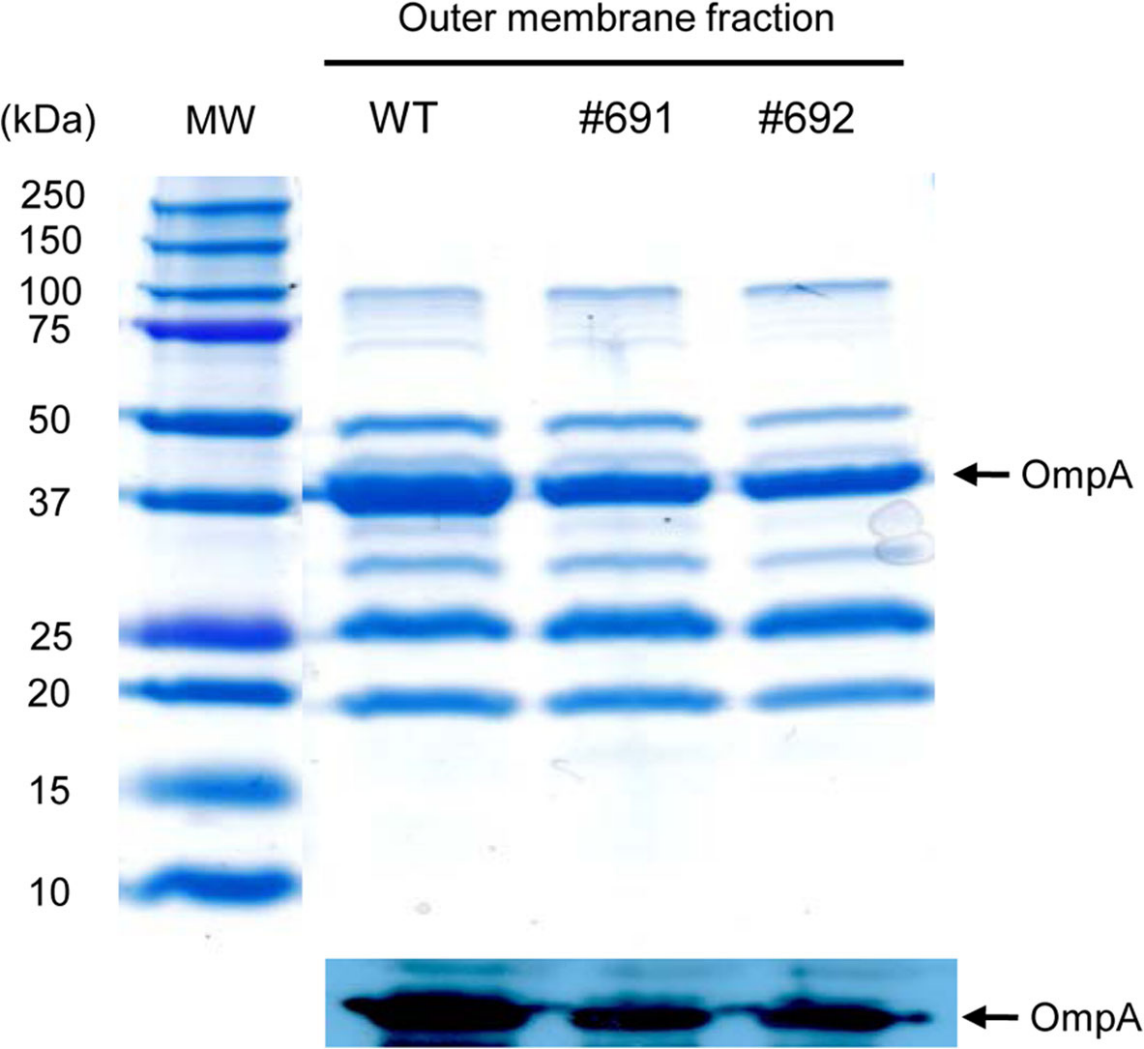
Production of OmpA in the outer membrane fraction of transposon-inserted *A. baumannii* mutant strains. Bacteria were cultured in LB broth for 24 h and proteins (10 μg) in the outer membrane fractions were separated on a 12% SDS-PAGE gel. MW, molecular weight marker; WT, *A. baumannii* ATCC 17978; #691 and #692 mutant strains, Transposon was inserted in the open reading frame of the A1S_0749 (*bfmS*) gene. Western blot analysis was performed to identify 38 kDa-OmpA. Protein samples resolved on 12% SDS-PAGE gel were transferred to nitrocellulose membranes and immunoblotted with a polyclonal anti-rabbit OmpA immune sera

To identify genes controlling OmpA production or localization in the outer membrane, random transposon mutagenesis was performed in *A. baumannii* ATCC 17978. The mutant library was screened for biofilm formation at an optical density of 570 nm (OD_570_), because Δ*ompA* mutant showed a significant reduction in biofilm formation [31]. Tn-inserted mutant strains that was inhibited ≥50% of biofilm formation compared with biofilm formation of the wild-type strain were then screened for OmpA production in the outer membrane using sodium dodecyl sulfate-polyacrylamide gel electrophoresis (SDS-PAGE) analysis. Two mutant strains (#691 and #692), in which Tn was inserted between nucleotide 954 and 955 in the A1S_0749 (*bfmS*) gene, exhibited low production of OmpA in the outer membrane as compared to wild-type *A. baumannii* ATCC 17978 (Fig. 1).

### Construction of the Δ*bfmS* mutant and its protein profile in the outer membrane

**Fig. 2:**
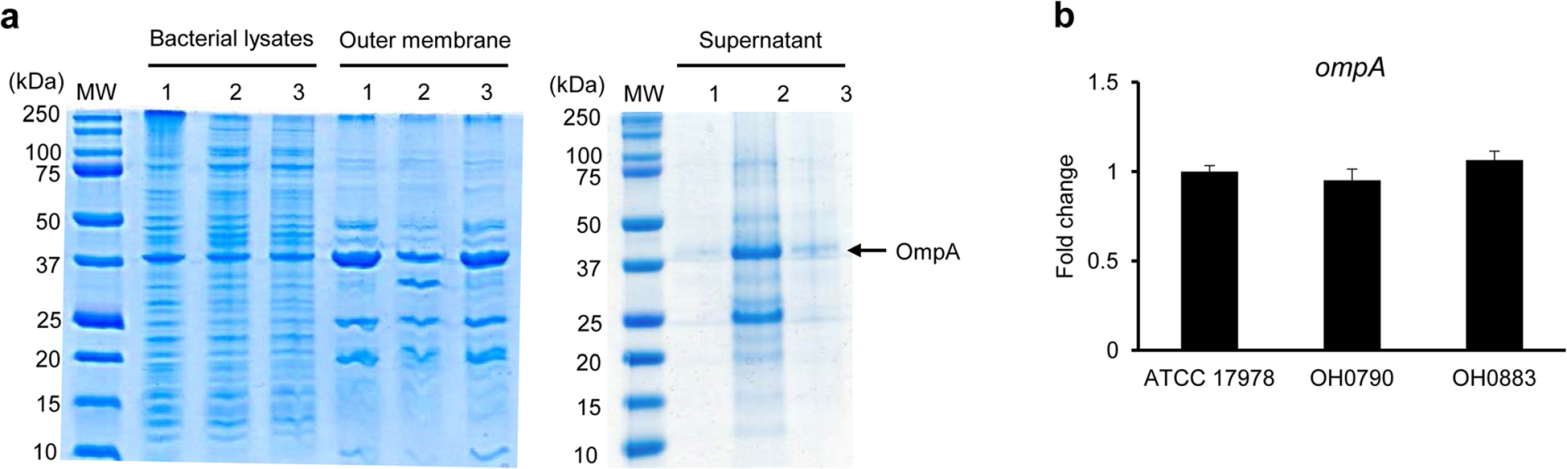
Production of OmpA protein and expression of *ompA* gene in *A. baumannii* strains. **a** SDS-PAGE analysis of bacterial proteins. The bacterial lysates and outer membrane fractions corresponding to 10 μg of protein were separated on a 12% SDS-PAGE gel. Proteins precipitated from the culture supernatants (200 ml) were resuspended in 200 μl of PBS and then 15 μl of the samples were separated on 12% SDS-PAGE gel. Lane MW, molecular weight marker; 1, *A. baumannii* ATCC 17978; 2, Δ*bfmS* mutant OH0790; 3, Δ*bfmS*-complemented OH0883. **b** Transcription levels of *ompA* in the three *A*. *baumannii* strains were determined using qPCR. The data are mean ± SD expression levels of the target gene in each strain relative to expression of this gene in *A*. *baumannii* ATCC 17978. Data were obtained from three independent experiments

To determine whether the Δ*bfmS* mutant increasingly released outer membrane proteins, including CarO and OmpA, in the supernatants as previously described [30], the Δ*bfmS* mutant (OH0790) of *A. baumannii* ATCC 17978 was constructed using a markerless gene deletion method [31]. The *bfmS*-complemented OH0883 strain was constructed (Table 1). SDS-PAGE analysis was performed in the wild-type, Δ*bfmS* mutant, and *bfmS*-complemented strains. Protein profiles in bacterial lysates were not different among the three *A. baumannii* strains (Fig. 2a). However, production of OmpA and ca. 33 kDa-sized proteins in the outer membrane was different between the wild-type and Δ*bfmS* mutant strains. The Δ*bfmS* mutant released more proteins, including OmpA and ca. 25 kDa-sized proteins, in the supernatants than the wild-type strain. The expression of *ompA* was not different among the wild-type, Δ*bfmS* mutant, and *bfmS*-complemented strains (Fig. 2b).

**Table 1:** Bacterial strains and plasmids used in this study

Bacteria or plasmids	Relevant characteristics^a^	Reference or source
Bacterial strains		
*A*. *baumannii*		
ATCC 17978	Wild-type strain	ATCC
OH0790	ATCC 17978 with Δ*bfmS*	This study
OH0883	*bfmS* rescue in OH0790	This study
HDK14	ATCC 17978 with Δ*ompA*	[31]
*E. coli*		
DH5α	*supE44 ΔlacU169 (Φ80 lacZ ΔM15) hsdR17 recA1 endA1 gyrA96 thi-1 relA1*; plasmid replication	[31]
S17-l λ pir	*λ-pir* lysogen; *thi pro hsdR hsdM*+ *recA* RP4–2 Tc::Mu-Km::*Tn*7; Tp^r^ Sm^r^; host for π-requiring plasmids; conjugal donor	[32]
Plasmids		
pRL27	Tn5-RL27; *ori*R6K; Km^r^	[33]
pBR322	Cloning vector; Ap^r^, Tc^r^	New England Biolabs
pUC4K	pUC4 with *nptI*; Ap^r^, Km^r^	Amersham Pharmacia Biotech
pHKD01	Suicide vector; *ori*R6K, *sacB*, and Cm^r^	[31]
pOH786	pHKD01 with Δ*bfmS*::*nptI*; Cm^r^, Km^r^	This study

^a^ *Tp*^*r*^ trimethoprim-resistant, *Sm*^*r*^ streptomycin-resistant, *Ap*^*r*^ ampicillin-resistant, *Km*^*r*^ kanamycin-resistant, *Cm*^*r*^ chloramphenicol-resistant, *Tc*^*r*^ tetracycline-resistant

### Phenotypic characteristics of the Δ*bfmS* mutant strain

**Fig. 3:**
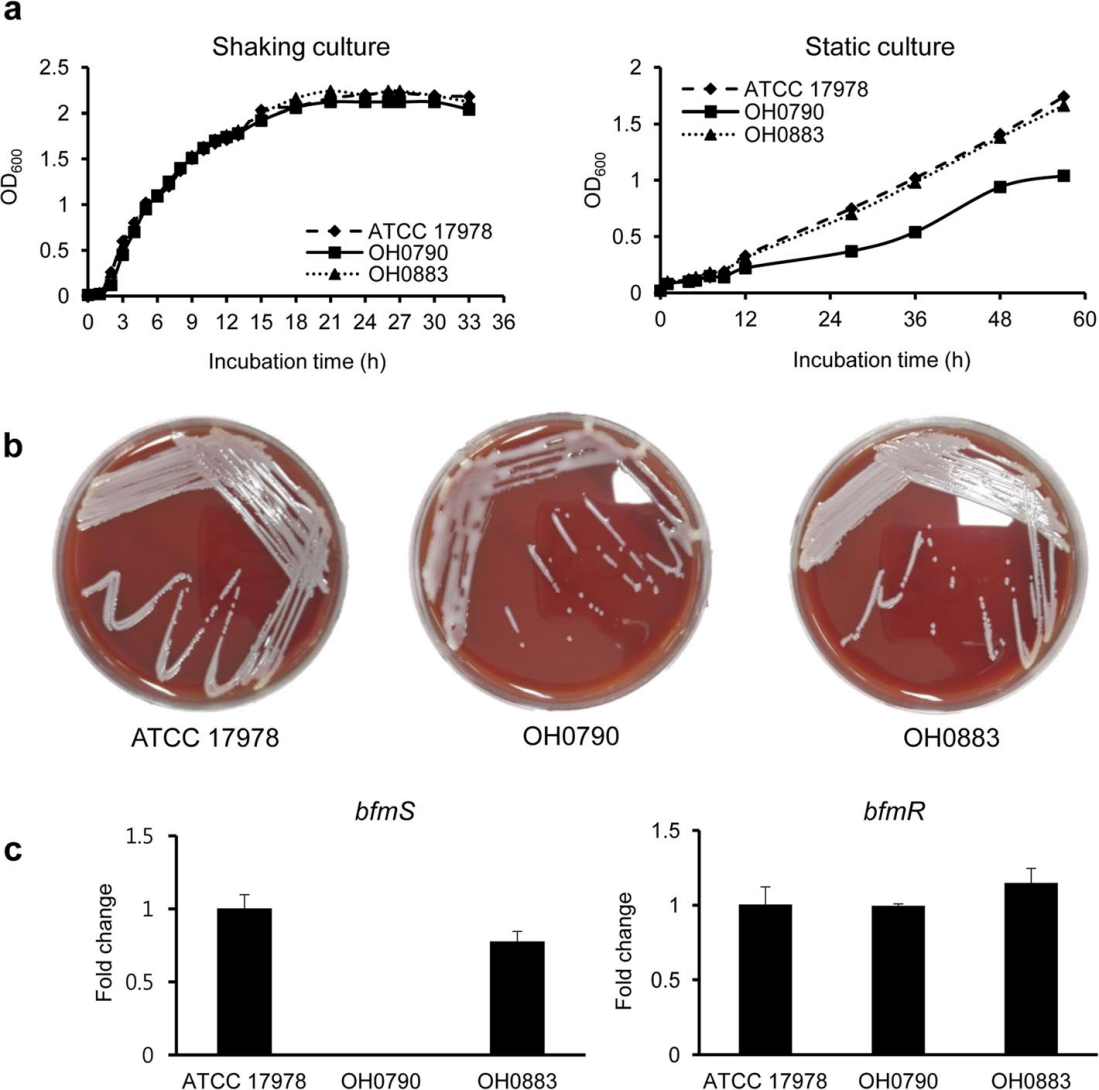
Characteristics of the Δ*bfmS* mutant strain. **a** *A. baumannii* strains were grown in LB broth under shaking or static conditions and then OD_600_ was determined at the indicated times. The data are representative of three experiments with similar results. **b** *A. baumannii* strains were cultured overnight on blood agar plates. **c** Transcription levels of *bfmS* and *bfmR* in *A*. *baumannii* strains were determined using qPCR. The data are mean ± SD expression levels of the target genes in each strain relative to expression of these genes in *A*. *baumannii* ATCC 17978. Data were obtained from three independent experiments

To determine whether *bfmS* affected the growth of *A. baumannii* strains, bacterial growth was measured at OD_600_. The growth rate was not different between the wild-type and Δ*bfmS* mutant strains cultured under shaking conditions, but growth retardation was observed in the Δ*bfmS* mutant cultured under static conditions (Fig. 3a). To investigate whether deletion of *bfmS* led to hyperproduction of exopolysaccharides as previously described [23], *A. baumannii* strains were cultured in blood agar plates for 24 h. The Δ*bfmS* mutant OH0790 was more viscous than the wild-type strain (Fig. 3b). Deletion of the *bfmS* gene did not alter the expression of *bfmR* in *A. baumannii* (Fig. 3c). Bacterial growth in static and shaking culture conditions, the production of exopolysaccharides, and the expression of *bfmS* were restored in the *bfmS*-complemented OH0883 strain.

### Effect of *bfmS* on pathogenic traits of *A. baumannii*

**Fig. 4:**
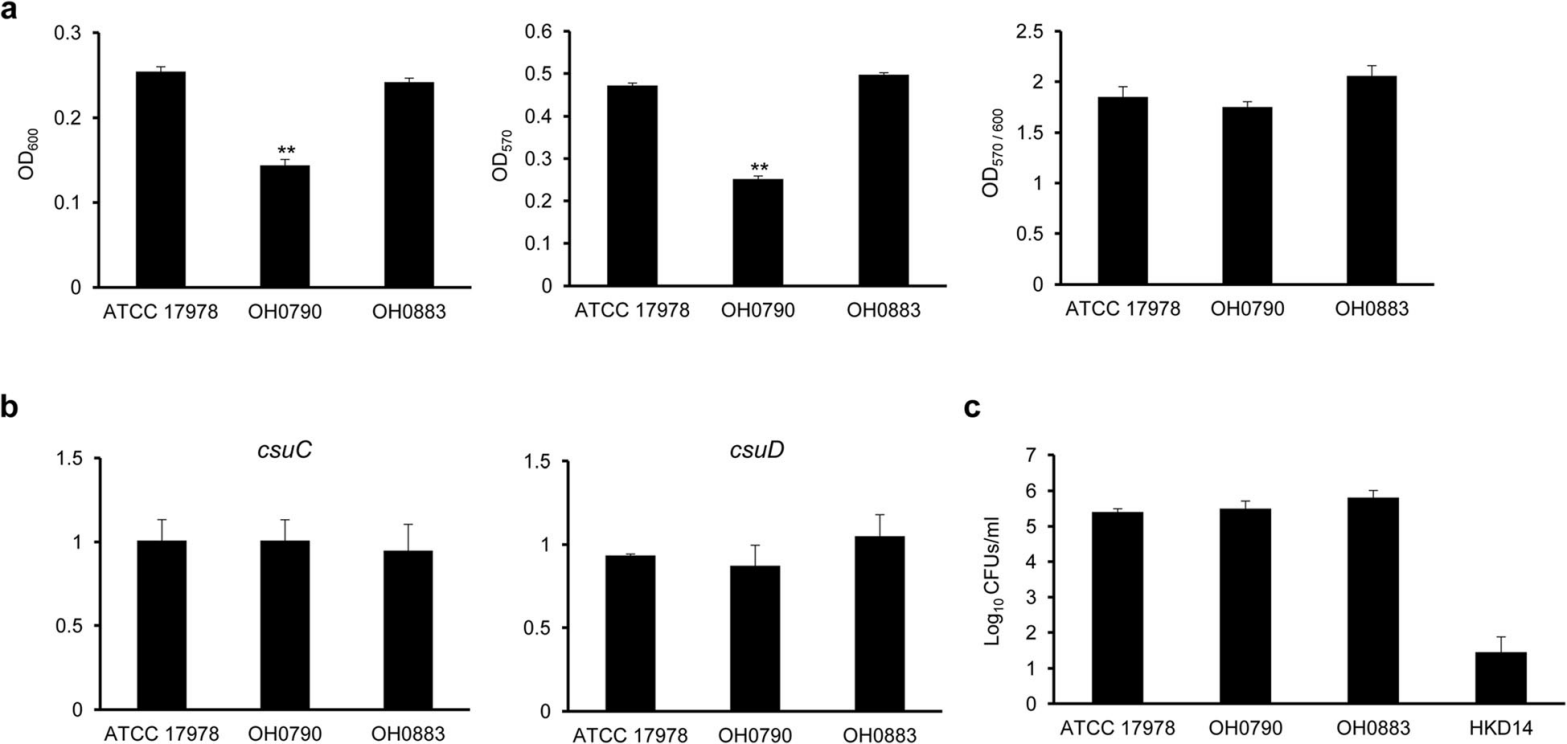
Biofilm formation, expression of the *csuCD* genes, and interactions with host cells in *A. baumannii* strains. **a** Biofilms formed on 5 ml polystyrene tubes were stained with crystal violet. The amount of crystal violet eluted from the biofilms with ethanol was quantified as the OD_570_ normalized to total bacterial growth (OD_600_). The data are presented as mean ± SD of three independent experiments. ** *p* < 0.01 compared to wild-type ATCC 17978. **b** Transcription levels of *csuC* and *csuD* in the three *A*. *baumannii* strains were determined using qPCR. Data are mean ± SD expression levels of the target genes in each strain relative to expression of these genes in *A*. *baumannii* ATCC 17978. The data were obtained from three independent experiments. **c** Adherence to and invasion of A549 cells by *A. baumannii* strains. A549 cells were infected with the *A. baumannii* strains at MOI 100 for 3 h, and then the cell monolayers were lysed with Triton X-100. Dilutions of the bacterial lysates were plated on LB agar, and CFUs were counted. The data are presented as mean ± SD of three independent experiments

To investigate the role of *bfmS* in pathogenic traits of *A. baumannii*, the ability of wild-type and Δ*bfmS* mutant strains to form biofilms on a polystyrene surface was determined. Bacterial growth at OD_600_ and biofilm cells at OD_570_ were significantly different between the wild-type and Δ*bfmS* mutant strains, respectively, but biofilm cells relative to planktonic and sessile cells (OD_570/600_) were not different between the two strains (Fig. 4a). Complementation of the *bfmS* gene deletion restored the wild-type biofilm formation phenotype. The expression of *csuC* and *csuD*, which are required for pili assembly and biofilm formation [24], was not different between the wild-type and Δ*bfmS* mutant strains (Fig. 4b). Next, to investigate the involvement of *bfmS* in adherence and invasion of host cells, A549 cells were infected with *A. baumannii* strains at multiplicity of infection (MOI) 100 for 3 h, and the number of bacteria adhered to and invading A549 cells was counted. No significant differences in numbers of bacteria were observed between wild-type (2.57 × 10^5^ colony forming units [CFUs]), Δ*bfmS* mutant (3.47 × 10^5^ CFUs), and *bfmS*-complemented (7.09 × 10^5^ CFUs) strains (Fig. 4c). The CFUs of Δ*ompA* mutant HKD14 were significantly decreased compared to the wild-type strain, as observed in a previous study [31].

### Effect of *bfmS* on the antimicrobial susceptibility of *A. baumannii*

Minimum inhibitory concentrations (MICs) of antimicrobial agents for the wild-type, Δ*bfmS* mutant, and *bfmS*-complemented strains were determined. The Δ*bfmS* mutant was more resistant to aztreonam (2.67-fold) and colistin (2.63-fold) than the wild-type strain (Table 2). The remaining antimicrobial agents tested showed the same or a < 2-fold difference in MICs for the Δ*bfmS* mutant. MICs of all antimicrobial agents determined for the *bfmS*-complemented strain were the same as, or similar to, those for the wild-type strain.

**Table 2:** MICs of antimicrobial agents for *A. baumannii* strains used in this study

Antimicrobial agent	MIC (μg/ml)		
	ATCC 17978	OH0790	OH0883
Aztreonam	24	64	32
Ceftazidime	4	6	4
Imipenem	0.19	0.25	0.19
Colistin	0.38	1	0.38
Ciprofloxacin	0.125	0.125	0.19
Nalidixic acid	3	3	4
Gentamicin	0.25	0.38	0.25
Tobramycin	0.25	0.38	0.25
Tetracycline	1.5	1.5	1.5
Tigecycline	0.125	0.125	0.125
Trimethoprim	> 32	> 32	> 32

### Effect of *bfmS* on OMV production

**Fig. 5:**
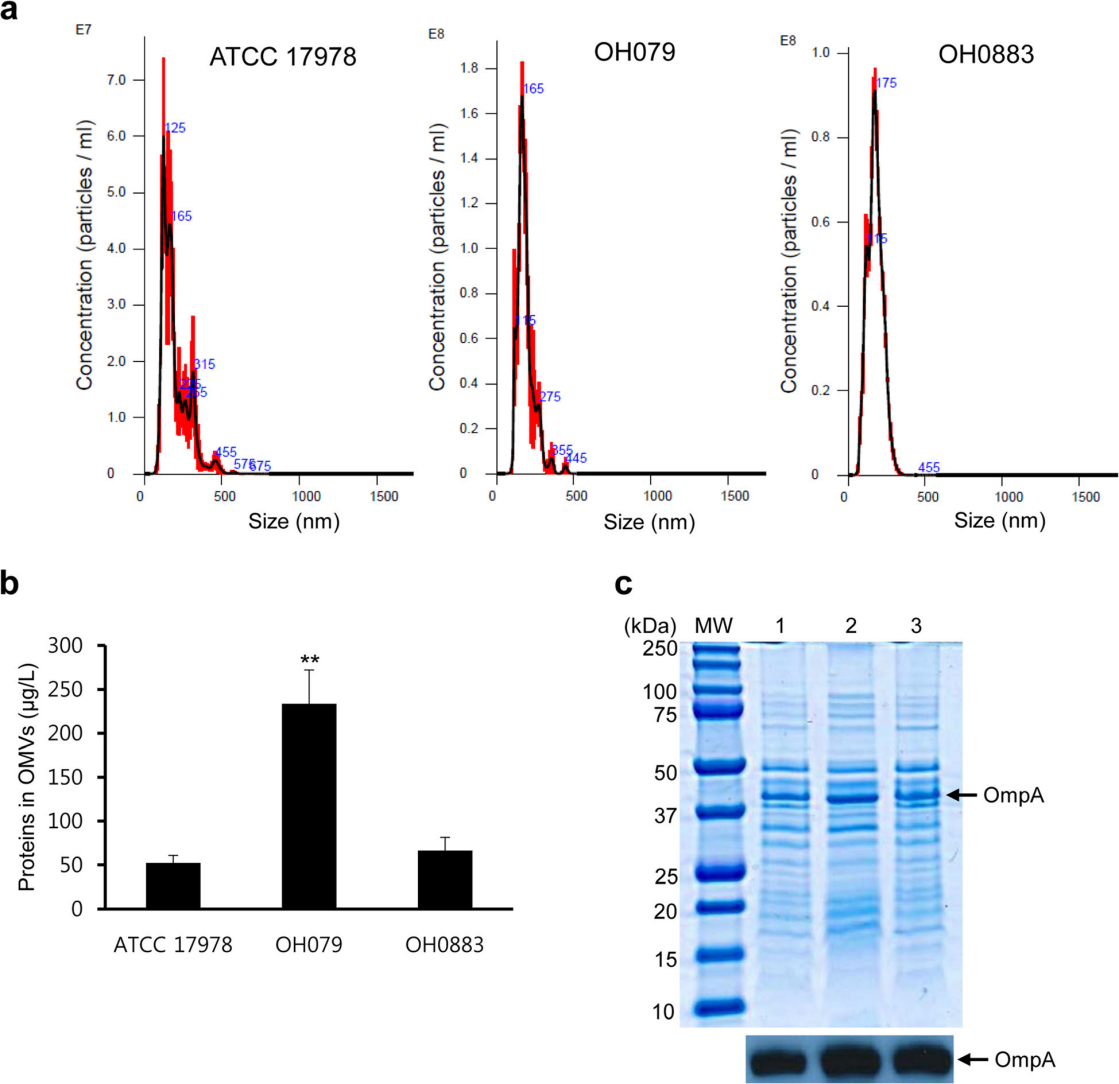
OMV production and its protein profile in *A. baumannii* strains. (**a** and **b**) Production of OMVs from *A. baumannii* strains. OMVs were isolated from *A. baumannii* cultured in LB broth. **a** The size and number of OMV particles isolated from three *A. baumannii* strains were determined using nanoparticle tracking analysis. The data are representative of three independent experiments with similar results. **b** The protein concentration of OMVs isolated from 1 L of bacterial culture was measured using a modified BCA assay. The data are presented as mean ± SD of two independent experiments. ** *p* < 0.01 compared to wild-type ATCC 17978. **c** SDS-PAGE and western blot analyses of OMV proteins. Protein samples were resolved by SDS-PAGE in 12% gels, transferred to nitrocellulose membranes, and immunoblotted with a polyclonal anti-rabbit OmpA immune sera. Lane MW, molecular weight marker; 1, *A. baumannii* ATCC 17978; 2, Δ*bfmS* mutant OH0790; 3, Δ*bfmS*-complemented OH0883

We determined OMV production in the Δ*bfmS* mutant, because a large amount of OmpA in the culture supernatants was packaged in *A. baumannii* OMVs [13]. *A. baumannii* strains were cultured in Luria-Bertani (LB) broth to reach late exponential phase and then OMVs were isolated from the culture supernatants. The sizes of OMVs from the wild-type, Δ*bfmS* mutant, and *bfmS*-complemented strains were 193.7 ± 11.9 nm, 186.8 ± 1.6 nm, and 174.8 ± 1.3 nm, respectively (Fig. 5a). OMV samples obtained from 1 L culture of the wild-type, Δ*bfmS* mutant, and *bfmS*-complemented strains contained 5.1 × 10^12^, 1.2 × 10^13^, and 8.4 × 10^12^ particles, respectively. The Δ*bfmS* mutant produced 4.46 (233.3 ± 38.7 μg/L) times more OMV proteins than the wild-type strain (52.3 ± 8.7 μg/L) (Fig. 5b). Further, SDS-PAGE analysis exhibited that protein profiles were very similar among the three different OMVs, but the intensity of several protein bands was different between OMVs from the wild-type and Δ*bfmS* mutant strains (Fig. 5c). Western blot analysis showed that OMVs derived from the Δ*bfmS* mutant contained more OmpA than those from the wild-type strain.

### Effect of *bfmS* on OMV-mediated pathogenesis of *A. baumannii*

**Fig. 6:**
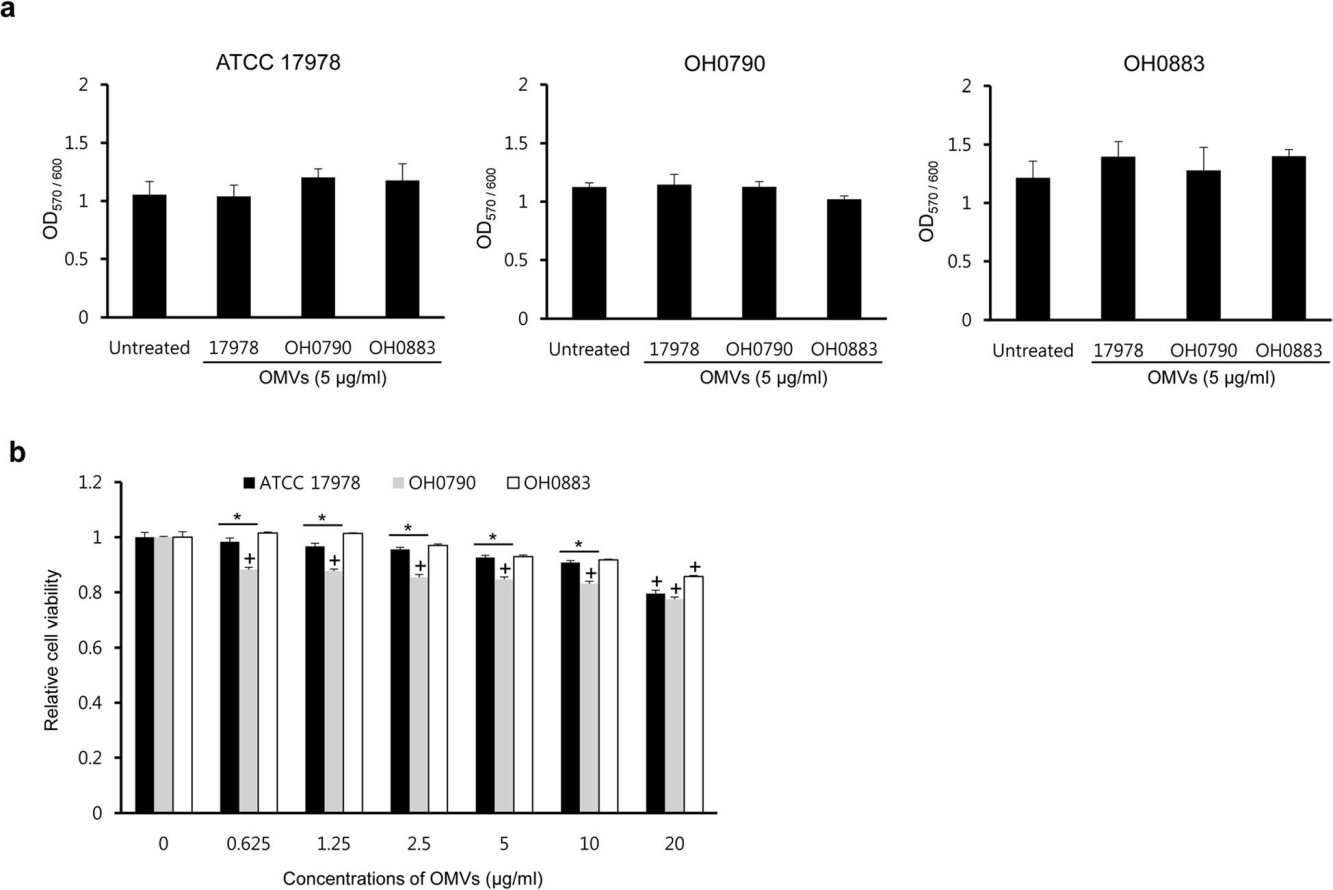
Pathogenic effect of OMVs derived from three *A. baumannii* strains. **a** Biofilm formation of *A. baumannii* strains cultured with OMVs from different *A. baumannii* strains. *A. baumannii* was inoculated in polystyrene tubes and then OMVs (5 μg/ml) obtained from different *A. baumannii* strains were added to bacterial culture media. Biofilms formed on polystyrene tubes were stained with crystal violet. The amount of crystal violet eluted from the biofilms with ethanol was quantified as the OD_570_ normalized to total bacterial growth (OD_600_). The data are presented as mean ± SD of three independent experiments. **b** Cytotoxicity of A549 cells treated with OMVs from *A. baumannii* strains. Cells were treated with various concentrations of *A. baumannii* OMVs for 24 h. Cell viability was determined using the MTT assay. Data are presented as mean ± SD of three independent experiments. + *p* < 0.05 compared to untreated control cells. * *p* < 0.05 comparing the same concentration of OMVs from *A. baumannii* ATCC 17978

To determine whether OMVs derived from the wild-type and Δ*bfmS* mutant strains played a different role in biofilm formation, OMVs (5 μg/ml) isolated from the wild-type, OH0790, and OH0883 strains were added to each bacterial culture after *A. baumannii* strains were inoculated in a polystyrene tube. Biofilm formation (OD_570/600_) was not significantly different between the wild-type and Δ*bfmS* mutant strains regarding the treatment of different OMVs (Fig. 6a). Next, we determined host cell cytotoxicity induced by OMVs isolated from the three *A. baumannii* strains, because OmpA in *A. baumannii* OMVs was responsible for the cytotoxicity of epithelial cells [13]. A549 cells were treated with various concentrations (0.625–20 μg/ml protein concentrations) of OMVs isolated from three *A. baumannii* strains for 24 h, and cell viability was assessed using the 3-[4,5-dimethylthiazol-2-yl]-2,5 diphenyltetrazolium bromide (MTT) assay. Cytotoxicity was induced in A549 cells treated with 20 μg/ml of the wild-type and *bfmS*-complemented strain OMVs, whereas cytotoxicity was induced in A549 cells treated with ≤0.625 μg/ml of the Δ*bfmS* mutant OMVs (Fig. 6b). Cytotoxicity significantly differed between the wild-type and Δ*bfmS* mutant OMVs at concentrations ≥0.625 μg/ml.

## Discussion

The response regulator BfmR contributes to the pathogenesis of *A. baumannii* infections regarding biofilm formation, antimicrobial resistance, and bacterial survival and disease process in animal models, whereas sensor kinase BfmS negatively regulates BfmR and plays a less relevant role [22–27]. The present study demonstrated that BfmS controlled OMV production. Moreover, BfmS negatively regulated resistance to aztreonam and colistin and OMV-mediated host cell cytotoxicity.

Two research groups characterized the role of BfmS in the pathogenicity of *A. baumannii* ATCC 17978 using BfmS-deficient mutant strains [22, 23, 30]. Liou et al. [30] showed that insertional inactivation of the *bfmS* gene in *A. baumannii* 17,978 resulted in reduction in biofilm formation, adherence to host cells, and survival in human serum. However, other studies conducted by Geisinger et al. [22, 23] demonstrated that the Δ*bfmS* mutant of *A. baumannii* 17,978 constructed by allelic replacements with the *aacC1* gene was resistant to killing by rabbit serum and was more virulent than the wild-type strain in a murine model of systemic infection. The discrepancy in virulence of *A. baumannii* ATCC 17978 mutants lacking BfmS between the two study groups was possibly due to different methods of mutant construction. We therefore constructed the Δ*bfmS* mutant of *A. baumannii* ATCC 17978 by markerless, in-frame deletions. Phenotypes of the Δ*bfmS* mutant constructed in this study, regarding bacterial growth under shaking culture conditions and hyperproduction of exopolysaccharides, were consistent with the previous studies [22, 23]. However, the Δ*bfmS* mutant constructed in this study exhibited growth retardation under static culture conditions. The ability to form biofilms (OD_570/600_) was not different between the wild-type and Δ*bfmS* mutant strains. The mutant of *A. baumannii* ATCC 17978 with *bfmS*::Tn showed a significant reduction in biofilm formation, which only measured biofilm cells by staining with crystal violet at OD_595_ [30], whereas the mutant derivative of *A. baumannii* ATCC 19606 with *bfmS*::Tn displayed no drastic defect in biofilm formation, which measured the biofilm cells relative to bacterial growth (OD_580/600_) [24]. The low ability to form biofilms in the *A. baumannii* ATCC 17978 mutant with *bfmS*::Tn conducted by Liou et al. [30] possibly resulted from growth retardation of this mutant strain under static culture conditions.

BfmS negatively regulates the production of capsular exopolysaccharides via phosphorylation of the cognate regulator BfmR [22]. In the present study, the Δ*bfmS* mutant showed hypermucoid phenotype as compared to the wild-type strain, but *bfmR* gene expression was not different between the two strains. The expression of *csuC* and *csuD* genes was not different between the wild-type and Δ*bfmS* mutant strains. RNA-sequencing analysis also showed that deletion of the *bfmS* gene did not significantly alter expression of the *bfmR* gene and *csuA/BABCDE* operon in *A. baumannii* ATCC 17978 [23]. The *csuA/BABCDE* operon plays a role in biofilm formation, but not in adherence to bronchial epithelial cells [24]. OmpA contributes to both biofilm formation and adherence to host cells [10, 31]. Adherence and invasion of *A. baumannii* in host cells were not different between the wild-type and Δ*bfmS* mutant strains, although the Δ*bfmS* mutant produced less OmpA in the outer membrane than the wild-type strain. Other bacterial molecules such as poly-β-(1,6)-N-acetyl glucosamine [34], a homolog of the staphylococcal biofilm-associated protein (BAP) [35], BAP-like proteins [36], and the products of *LHp2_11085* gene [37] may compensate biofilm formation and host cell adherence of the Δ*bfmS* mutant. Taken together, our results suggest that deletion of *bfmS* increases the production of capsular exopolysaccharides but does not affect biofilm formation and adherence and invasion of *A. baumannii* ATCC 17978 in host cells.

The Δ*bfmS* mutant produced more OMV particles than the wild-type strain. Moreover, the Δ*bfmS* mutant released more proteins, including OmpA, via OMVs in the supernatants. Instead, the Δ*bfmS* mutant produced less OmpA in the outer membrane than the wild-type strain. Although the biogenesis of OMVs was not fully understood, several models of OMV biogenesis were proposed, such as a reduction in cross-linking between the outer membrane and peptidoglycans [38], accumulation of phospholipids in the outer leaflet of the outer membrane [39], and deacylation of lipopolysaccharides [40]. We previously showed that the Δ*ompA* mutant of *A. baumannii* ATCC 19606 produced 13.2 times more OMV proteins and 7.30 times more OMV lipopolysaccharides than the wild-type strain [41]. These results suggest that OmpA directly or indirectly contributes to the production of *A. baumannii* OMVs. OmpA interacts with other membrane proteins in the outer and inner membranes and peptidoglycans [42, 43]. The C-terminal OmpA-like domain of OmpA interacts with diaminopimelate of peptidoglycan [43]. Therefore, low localization of OmpA in the outer membrane reduces interaction of the outer membrane with peptidoglycan, which may increase OMV production. The association of bacterial extracellular vesicle production with TCSs was reported in *Streptococcus pyogenes* [44]. Inactivating mutations in sensor kinase (CovS) of control of virulence regulator-sensor (CovRS) increased extracellular vesicle production in *S. pyogenes*. Moreover, mutant strains expressing truncated and inactive CovS produced a significantly higher number of extracellular vesicles relative to the wild-type strain. Although the association of OMV biogenesis with TCSs, especially in sensor kinases, has not been characterized in gram-negative bacteria, genes under the control of BfmS or BfmRS may regulate OMV biogenesis. The exact mechanisms by which BfmS controls OMV production should be determined in further studies.

OMVs derived from the Δ*bfmS* mutant were more cytotoxic in cultured epithelial cells than OMVs from the wild-type strain. We previously showed that several virulence factors, including OmpA, β-lactamases, and tissue-degrading enzymes, were associated with OMVs of *A. baumannii* ATCC 19606 [13, 45]. The OMVs derived from *A. baumannii* ATCC 19606 induced host cell death, whereas OMVs from the Δ*ompA* mutant did not [13], thus suggesting that OmpA in OMVs is directly responsible for host cell cytotoxicity. In this study, the Δ*bfmS* mutant rather than wild-type strain showed a reduced susceptibility to colistin and aztreonam. Hyperproduction of exopolysaccharides in the Δ*bfmS* mutant may explain reduced susceptibility to colistin [26]. The Δ*bfmS* mutant of *A. baumannii* 17,978 constructed by allelic replacements with the *aacC1* gene also showed a reduced susceptibility to aminoglycosides (amikacin) and β-lactams (mecillinam, ampicillin, carbenicillin, cephalexin, aztreonam, ceftazidime, and sulbactam), whereas the Δ*bfmRS* mutant showed hypersensitivity to several classes of antimicrobial agents, including aminoglycosides and β-lactams [23]. The BfmRS system controls antimicrobial resistance via cell wall homeostasis, and BfmS negatively regulates the resistance activity of BfmR.

## Conclusions

The BfmRS system regulates the physiology and pathogenic traits of *A. baumannii*. However, the role of BfmS in the pathogenic traits of *A. baumannii* is still poorly understood. Here, we demonstrate that BfmS controls production of OMVs and regulates antimicrobial resistance and OMV-mediated host cell cytotoxicity. Understanding of the BfmRS-mediated regulatory system is expected to provide insights into *A. baumannii* pathogenicity. Controlling the BfmS may represent a strategy to combat this notorious pathogen, because overproduction of OmpA in *A. baumannii* is a risk factor for nosocomial pneumonia, bacteremia, and high mortality rate [46].

## Methods

### Bacterial strains, plasmids, and growth conditions

The bacterial strains and plasmids used in this study are listed in Table 1. *A. baumannii* ATCC 17978 was purchased from American Type Culture Collection (ATCC). *Escherichia coli* DH5α (Catalogue number 18258012) was purchased from Invitrogen (Grand Island, NY, USA). Bacteria were grown in LB medium at 37 °C. *A. baumannii* strains were cultured in blood agar plates containing 5% sheep red blood cells for the analysis of viscosity of bacterial colonies. Chloramphenicol (20 μg/ml) or kanamycin (50 μg/ml) was added to the growth medium to maintain plasmids in *E. coli*. *A. baumannii* merodiploids were selected on medium supplemented with kanamycin (30 μg/ml) and ampicillin (100 μg/ml).

### Random transposon mutagenesis

A mutant library of *A. baumannii* was constructed by random transposon mutagenesis. *A. baumannii* ATCC 17978 was mutagenized using the S17–1 λ *pir tra* strain [32] containing pRL27, a suicide vector carrying the transposable mini-*Tn*5 element [33]. *Tn*-inserted colonies were selected by plating on LB agar plates containing 50 μg/ml kanamycin and stored at − 80 °C until use. To determine transposon insertion sites on the bacterial genome, bacterial genomic DNA was digested by *Bam*HI. The digested DNA was ligated with *Bam*HI-digested pBR322 (Catalogue number N3033 L, New England Biolabs, Ipswich, MA, USA) and then introduced into *E. coli* DH5α. The transposon insertion site was analyzed by DNA sequencing.

### Construction of the Δ*bfmS* mutant strain

The *bfmS* (A1S_0749) gene of *A. baumannii* ATCC 17978 was deleted by an overlap extension polymerase chain reaction (PCR) method as previously described [31]. The genomic DNAs purified from *A*. *baumannii* strains and pUC4K (Catalogue number 27–4958-01, Amersham Pharmacia Biotech, Piscataway, NJ, USA) for amplification of the kanamycin resistance cassette were used as templates for the PCR. In brief, a mutated DNA fragment, in which upstream and downstream regions of the *bfmS* gene were combined with *npt*I conferring kanamycin resistance by overlap extension PCR using specific primers (Table 3), was ligated into *Fsp*I-digested pHKD01 to generate pOH786 (Table 1). *E. coli* S17–1 λ *pir* strain containing pOH786 was used as a conjugal donor to *A. baumannii* ATCC 17978. Conjugation and isolation of the transconjugants were performed as previously described [31]. Deletion of the *bfmS* gene in *A. baumannii* ATCC 17978 was confirmed by PCR analysis and the Δ*bfmS* mutant was named OH0790 (Table 1).

**Table 3:** Oligonucleotides used in this study

Primers	Oligonucleotide sequence (5′ → 3′)^a^	Use
Deletion of *bfmS* in *A. baumannii* ATCC 17978		
BFMS01F	ATCAGTTTGGTGAACGCCTACTT	Amplification of upstream region of *bfmS*
BFMS01R	AATAAAAAAAGCACCATCAGATGCGTCAGAAATCCAA	
BFMS02F	GGTGCTTTTTTTATTGCTTCATTTAT	Amplification of downstream region of *bfmS*
BFMS02R	CTTCACGAGGCAGACCGCCACTTACCGTTTCCAGTAT	
Single-copy complementation of *bfmS* in *A. baumannii* Δ*bfmS* mutant		
BFMS03F	GATCATTATTAAGGCAATCTGATTAAACTTCCTATAAGGTTGG	Amplification of *bfmS* coding region with its native promoter
BFMR03R	ATTAAAGCAGGTGATATGAAGCAATAAAAAAAGCACCTT	
ABaTn01F	TGGTTTGAGCAATTGACTTGG	Amplification of the upstream region of *att*Tn7
ABaTn01R	GCCTTAATAATGATCTTTTTTGAATTACT	
ABaTn02F	ATCACCTGCTTTAATAATTGATTGATTA	Amplification of the downstream region of *att*Tn7
ABaTn02R	GCAACACCTTCTTCACGAGGCAGACAGTCGGTTTTAGCAGACCGTAC	
Amplification of kanamycin-resistance cassette		
U1	GTCTGCCTCGTGAAGAAGGTG	Amplification of *npt*I
U2	GATCCGTCGACCTGCAGG	

^a^ Underlined sequences indicate regions that are not complementary to the templates

### Complementation of the *bfmS* gene in the Δ*bfmS* mutant strain

To complement the *bfmS* mutation, the *bfmS* coding region with its native promoter was inserted into the *att*Tn7 site located downstream of the *glmS* gene in the genome of *A. baumannii* ATCC 17978 using the modified markerless gene deletion method [31]. A DNA fragment, in which the *bfmS* coding region with its native promoter and the upstream and downstream regions of the *att*Tn7 site were fused with *nptI* by overlap extension PCR using specifi14primers (Table 3), was cloned into *Fsp*I-digested pHKD01 to generate pOH875 (Table 1). The chimeric plasmid was integrated into the chromosome of the Δ*bfmS* mutant by conjugation-based gene transfer and homologous recombination. Insertion of the *bfmS* coding region with its native promoter was confirmed by PCR analysis. The *bfmS*-complemented strain was named OH0883 (Table 1).

### Isolation of OMVs

OMVs of *A. baumannii* strains were prepared from bacterial culture supernatants as previously described [13, 47]. Bacteria were cultured with 500 ml of LB broth with shaking at 37 °C until to reach late exponential phase (OD_600_ of 1.5). Bacterial cells were harvested by centrifugation at 8000 *g* for 15 min, and supernatants were filtered using a bottle-top filter with a 0.22 μm membrane. The filtered supernatants were concentrated using a QuixStand Benchtop System (GE Healthcare, Amersham, UK) with a 500 kDa hollow fiber membrane (GE Healthcare). OMV samples were collected by ultracentrifugation at 150,000 *g* at 4 °C for 3 h and then washed in phosphate-buffered saline (PBS) followed by another ultracentrifugation. The OMV fractions were then resuspended in PBS. The protein concentration of OMVs was determined using a modified bicinchoninic acid (BCA) assay (Thermo Scientific, Waltham, MA, USA). The purified OMVs were streaked on blood agar plates to check for sterility and then stored at -80 °C until use.

### SDS-PAGE and western blotting

Bacteria were cultured in LB broth with shaking at 37 °C until to reach 1.5 at OD_600_. Cultured bacterial cells were harvested and lysed by sonication (Branson Ultrasonics Corp., Danbury, CT, USA). After centrifugation at 1700 *g* for 20 min, the supernatant was centrifuged at 100,000 *g* for 1 h at 4 °C. The pellet containing cell envelope was resuspended in 10 mM HEPES buffer with 2% sodium lauryl sarcosine and incubated for 30 min at room temperature to solubilize the inner membrane. Then the suspension was centrifuged at 100,000 *g* for 1 h at 4 °C and outer membrane fractions were resuspended in PBS. Proteins in the culture supernatants (200 ml) were precipitated with 80% ammonium sulfate and then 10% trichloroacetic acid, and the samples were resuspended in 200 μl of PBS. The bacterial lysate, outer membrane fractions, and purified OMVs corresponding to 10 μg of protein were resuspended in SDS-PAGE sample buffer (1 M Tris HCl [pH 6.8], 10% SDS, 1% bromophenol blue, glycerol, and β-mercaptoethanol) and boiled for 10 min. Precipitated proteins (15 μl) in the culture supernatants were resuspended in SDS-PAGE sample buffer. The proteins were separated on a 12% SDS-PAGE gel, and gels were stained with Coomassie brilliant blue R-250 (Bio-Rad, Hercules, CA, USA). Western blot analysis was performed following SDS-PAGE. Proteins were electroblotted onto nitrocellulose membrane. Membranes were incubated with a polyclonal anti-rabbit OmpA immune serum. The membrane was incubated with a secondary antibody coupled to horseradish peroxidase and developed using an enhanced chemiluminescence system (Amersham Pharmacia Biotech).

### Nanoparticle tracking analysis (NTA)

OMV size and concentration were measured using a NanoSight NS500 instrument with a 488 nm laser module and sCMOS camera module (Malvern Instruments, Worcestershire, UK) [48]. Briefly, OMV samples were diluted in MilliQ water to a concentration of approximately 8–9 × 10^8^ particles/ml; the NTA measurement yielded 50–100 particles per frame. Samples were loaded in the sample chamber and videos were recorded for 30s three times. The captured data were analysed using NTA 3.1 software build 3.1.46. All measurements were performed in triplicate at room temperature.

### Bacterial growth studies

Overnight cultures of *A. baumannii* strains were diluted 1:20 in LB broth and cultured under shaking or static conditions for 36 and 60 h at 37 °C, respectively. Bacteria were sampled at the indicated times, and OD_600_ was determined. Bacterial growth was determined in triplicate.

### Biofilm assay

A biofilm formation assay was performed as previously described [14]. Overnight cultures were adjusted to an OD_600_ of 2.0, and diluted 200-fold in LB medium without sodium chloride. Aliquots (2 ml) of the bacterial suspension were inoculated into 5 ml polystyrene tubes and incubated without shaking at 37 °C for 24 h. Planktonic cells were removed, and the tubes were washed twice with 1 ml of PBS. Biofilm cells on the tube wall were stained with 0.1% w/v crystal violet solution for 15 min at room temperature. Then, biofilm formation was quantified using a biofilm cell-associated dye, which was eluted with 100% ethanol, as the absorbance at OD_570_, which was normalized to bacterial growth at OD_600_. To evaluate whether OMVs derived from *A. baumannii* strains affected biofilm formation, OMVs (5 μg/ml) were added to the bacterial culture after inoculation of bacteria in the tubes. Biofilm formation ability of the Tn-inserted *A. baumannii* mutants was determined using 96-well cell culture plates. A total of 200 μl of the bacterial suspension was incubated in U-bottomed 96-well microtiter plates at 37 °C for 24 h. In each plate, the wild-type strain was included as a control. Biofilm assays were performed in duplicate and repeated three times.

### Antimicrobial susceptibility test

MICs were determined by the Etest method according to the manufacturer’s instructions. Antimicrobial agents included aztreonam, ceftazidime, ciprofloxacin, colistin, gentamicin, imipenem, nalidixic acid, tetracycline, tigecycline, tobramycin, and trimethoprim (bioMe’rieux, Marcy-l’_Etoile, France). *E. coli* ATCC 25922 and *Pseudomonas aeruginosa* ATCC 27853 were used as quality control strains. Interpretation of antimicrobial susceptibility was based on guidelines of the Clinical Laboratory Standards Institute (CLSI) [49].

### RNA isolation and quantitative PCR

The mRNA expression levels of *bfmR*, *bfmS*, *ompA*, *csuC*, and *csuD* genes were analyzed. Bacteria were cultured to an OD_600_ of 1.5 in LB broth with shaking at 37 °C. Total RNA was extracted using the RNeasy Mini Kit (Qiagen, Valencia, CA, USA) according to the manufacturer’s instructions. Complementary DNA was generated by reverse transcription of 2 μg of total RNA using oligo dT primers and M-MLV reverse transcriptase in a total reaction volume of 20 μl (Enzynomics, Daejeon, Korea). The specific primers for *csuC* and *csuD* genes were described in previous studies [14]. The primer sequences were 5′-GTT TAA CCG TTT GTC GTG-3′ and 5′-GTG GTT GAA CTG GTT TCG-3′ for *bfmR*, 5′-TTG AAC TTA TTC ACC GCC TTT-3′ and 5′-GCC CGT AAT CCG AAC TTT GTT-3′ for *bfmS*, and 5′- TTG CAC TTG CTA CTA TGC TTG TTG-3′ and 5′- TGG CTG TCT TGG AAA GTG TAA CC-3′ for *ompA*. Gene transcripts were quantified using TOPreal™ qPCR 2X PreMIX (SYBR Green with high ROX) (Enzynomics) with an ABI PRISM 7500 Real-Time System (Applied Biosystems, Foster City, CA, USA) according to the manufacturer’s instructions. The amplification specificity was evaluated using melting curve analysis. Gene expression was normalized to 16S rRNA expression in each sample, and the fold change was determined using the ΔΔCt method. Gene expression assays were performed in three independent experiments.

### Cell culture

Human lung epithelial A549 cells were used to analyze interactions with bacteria or OMVs. A549 cells were obtained from the Korean Cell Line Bank (Seoul, Korea). A549 cells were grown in RPMI 1640 medium (HyClone, Logan, UT, USA) supplemented with 10% fetal bovine serum (HyClone), 2.0 mM _L_-glutamine, 100 U/ml penicillin, and 50 mg/ml streptomycin at 37 °C in 5% CO_2_. Confluent cells were seeded in 24- and 96-well plates for bacterial adherence and cell viability assays, respectively.

### Adherence and invasion assays

Adherence and invasion of A549 cells by *A. baumannii* strains were determined as previously described [10]. A549 cells were seeded at a density of 6 × 10^4^ cells in 24-well culture dishes. Cells were infected with *A. baumannii* strains at MOI 100 for 3 h. The infected monolayers were washed five times with PBS and then lysed with 0.1% Triton X-100 at 37 °C for 20 min. Dilutions of the lysates were plated on LB agar, and colonies were enumerated after 20 h of incubation. CFUs of the Δ*bfmS* mutant were compared with those of the wild-type and Δ*ompA* mutant strain (HKD14) of *A. baumannii* ATCC 17978 as the positive and negative controls, respectively. Adherence and invasion assays were performed in three independent experiments.

### Cell viability test

The viability of A549 cells was measured using the MTT assay (Abcam, Cambridge, UK). Cells were seeded at a concentration of 2 × 10^4^/well in a 96-well microplate. After treatment with different concentrations of *A. baumannii* OMVs for 24 h, cell viability was measured 3 h after treatment with MTT reagent at 600 nm. The cell viability assay was performed in three independent experiments.

### Statistical analysis

Data were analyzed using R 3.3.4 (https://www.r-project.org/). One-way analysis of variance (ANOVA) and Student’s t-tests were performed and post-hoc tests were applied when needed. Differences of *p* < 0.05 were considered statistically significant.

## Data Availability

All data generated or analysed during this study are included in this published article.

## Abbreviations

Ap^r^: Ampicillin-resistant
CFUs: Colony forming units
Cm^r^: Chloramphenicol-resistant
Km^r^: Kanamycin-resistant
MICs: Minimum inhibitory concentrations
MOI: Multiplicity of infection
OmpA: Outer membrane protein A;
OMVs: Outer membrane vesicles
Sm^r^: Streptomycin-resistant
Tc^r^: Tetracycline-resistant
TCSs: Two-component systems
Tp^r^: Trimethoprim-resistant
